# Health Promotion Interventions: Lessons from the Transfer of Good Practices in CHRODIS-PLUS

**DOI:** 10.3390/ijerph17041281

**Published:** 2020-02-17

**Authors:** Andrew Barnfield, Nella Savolainen, Anne Lounamaa

**Affiliations:** 1 EuroHealthNet, 1000 Brussels, Belgium; 2 National Institute for Health and Welfare, FI-00271 Helsinki, Finland; anne.lounamaa@thl.fi

**Keywords:** health promotion, disease prevention, intervention, implementation, good practice, children, adults, transfer

## Abstract

Health promotion and disease prevention often take the form of population- and individual-based interventions that aim to reduce the burden of disease and associated risk factors. There is a wealth of programs, policies, and procedures that have been proven to work in a specific context with potential to improve the lives and quality of life for many people. However, the challenge facing health promotion is how to transfer recognized good practices from one context to another. We present findings from the use of the implementation framework developed in the Joint Action project CHRODIS-PLUS to support the transfer of health promotion interventions for children’s health and older adults identified previously as good practices. We explore the contextual success factors and barriers in the use of an implementation framework in local contexts and the protocol for supporting the implementation. The paper concludes by discussing the key learning points and the development of the next steps for successful transfer of health promotion interventions.

## 1. Introduction 

Health promoters are well aware of the challenges faced by people with chronic disease, as well as the health systems’ efforts to meet these challenges [1]. In the European Union (EU) in 2016, two-thirds of early deaths of people under 75 were avoidable [2], i.e., 1.2 million out of 1.7 million deaths. Of those, 741,000 deaths could have been avoided through effective public health and primary prevention interventions, and 422,000 deaths could have been avoided through timely and effective healthcare interventions. In addition, 80% of healthcare costs are spent on chronic disease while only 3% of healthcare expenditure is assigned to chronic disease prevention [3]. 

In response to these challenges, the European Commission and Member States funded the CHRODIS-PLUS Joint Action. Joint Actions are a funding instrument under the third EU Health Program 2014–2020. They are designed and financed by Member State authorities and the EU to address specific priorities under the EU Health Program. The goal of CHRODIS-PLUS is to support Member States through cross-national initiatives identified in CHRODIS Joint Action (2014–2017) to reduce the burden of chronic disease, while assuring health systems sustainability and responsiveness. CHRODIS-PLUS aims to promote the implementation of recognized good practices with demonstrated success in different contexts. 

Joint Action (JA) CHRODIS-PLUS is a three-year initiative funded by the European Commission that started in 2017. Working with 22 partners from 14 EU Member States, the health promotion and primary prevention area aims to improve the knowledge and practice on health promotion and disease prevention. It builds on the successful results from the previous Joint Action CHRODIS that identified good practices. This study is part of the health promotion and primary prevention work package in CHRODIS-PLUS. The aim is to examine the possibility of implementing good practices identified in CHRODIS in different contexts with the aid of the implementation strategy, designed especially for CHRODIS-PLUS. 

The specific aim of CHRODIS-PLUS is to promote the implementation in several countries of innovative practices for patient empowerment, health promotion, and primary prevention, as well as quality management of chronic disease. In this paper, we focus on cross-national transfer of two good practices, Active School Flag and multimodal training. Both represent practices that promote physical activity and seek to change the environments and the motivation of the target group. 

A recognized good practice within CHRODIS-PLUS is based upon work carried out in JA CHRODIS. This involved more than 30 organizations from 13 EU Member States to identify 41 promising interventions and policies on health promotion and chronic disease prevention based on a jointly developed set of criteria. The identified interventions fed into a “Platform for Knowledge Exchange”, an up-to-date repository of good practices for disease prevention and chronic care stakeholders. The JA CHRODIS defined a “good practice” in accordance with the definition by the Food and Agricultural Organization of the United Nations: “*A good practice is not only a practice that is good, but a practice that has been proven to work well and produce good results, and is therefore recommended as a model. It is a successful experience, which has been tested and validated, in the broad sense, which has been repeated and deserves to be shared so that a greater number of people can adopt it*.” Each Member State partner identified and documented three or more highly promising and/or evidence-based practices with the collaboration of relevant ministries, institutes, and civil-society institutions. Special attention was given to practices with a focus on vulnerable populations and that were shown to have a positive impact on the health or health behavior of the target population.

An issue within efforts to reduce the burden of chronic disease is that there is a wealth of knowledge of what works but a lack of knowledge on how to implement good practices and programs [4,5]. The World Health Organization (WHO) Commission on the Social Determinants of Health highlighted effective interventions to improve the health of populations and to establish health equity [6]. To improve public health, evidenced informed policies, programs, and interventions need to be successfully implemented into routine practice by organizations in the community.

Implementation science is the field of work that attempts to solve a wide range of implementation problems. Its origins are in several disciplines and research traditions [7]. The field of implementation science is continually developing. However, the field needs to develop further to provide information about how to effectively implement health policies, programs, and practices that were proven to work [8]. Implementation science attempts to comprehend and work within real world conditions, instead of controlling for these conditions or to remove their influence as causal effects [9].

An important element for implementing good practices is context [10]. Context includes the social, cultural, economic, political, legal, and physical environment, as well as the institutional setting, comprising various stakeholders and their interactions, demographics, and epidemiological conditions [11,12]. However, there is an insufficient understanding of context and implementation, which contributes to a significant gap between research, practice, and transfer [10]. This is due to the fact that many interventions are complex and usually comprise multiple components. These components can act independently or interdependently, with the vital element for success being difficult to specify [9]. 

In 2016, the WHO affirmed that the interplay between an intervention for chronic disease and its local context can affect successful implementation [13]. However, the quality of reporting contextual factors is often weak when analyzing implementation [14]. Furthermore, the failure to capture context in appropriate ways manifests a major barrier to appraising transferability and applicability of implementing potentially relevant practices [15]. Implementation and context are inextricably linked, and interactions take place between them that challenge the transfer of an intervention into a new setting. [16] Therefore, it is vital to identify and understand the role of context and develop implementation strategies with which to mitigate contextual challenges.

Another key element in implementation research is the focus on working with actual populations that will be affected by an intervention, in contrast to selecting beneficiaries who may not represent the target population of an intervention as is often the case with randomized controlled trials [17]. In addition, the composition and configuration of health systems and their delivery are particularly important for implementation research on health [18]. 

Therefore, implementation research has many challenges for health promotion [19]. This is because of the many factors of success involved and the long-term reporting period required to establish what is working, for when, and for whom. The aim of this paper is to add to the knowledge base of implementation research by exploring the transfer of practices. This is via an implementation framework developed in CHRODIS-PLUS Joint Action for health promotion interventions for children and older adults. This paper makes an important contribution to existing knowledge on understanding contextual factors that are critical to successful implementation and how to transfer a good practice with an implementation framework from one context to another. 

The paper is set out as follows: firstly, we briefly describe the methodology used in implementation of the good practices in CHRODIS-PLUS. Secondly, we detail two examples from CHRODIS-PLUS work in health promotion on good practices and how they applied the implementation strategy. Thirdly, we describe the contextual success factors and barriers to the transfer and implementation of a good practice for health promotion. Finally, we conclude by outlining the key learning points from the implemented programs and the development of the next steps for successful transfer of health promotion interventions.

## 2. Methods and Materials

The implementation framework in CHRODIS-PLUS consists of the implementation strategy and the protocol for supporting the use of the implementation strategy.

The implementation strategy in CHRODIS-PLUS consists of three phases. The preparatory phase operated through September 2017 to August 2018 and included situation analyses, feasibility assessments, and getting the pilot action plans ready. The implementation phase, which recently ended, operated from September 2018 to January 2020. In the implementation phase, implementing sites monitored the progress of implementations and collected data. During the post-implementation stage, the implementers of the good practice will evaluate if the implementation was successful or not and report the experiences from the whole project. It will operate from February 2020 to September 2020.

The protocol for supporting the use of the implementation strategy was specially designed during the preparatory phase by the executive board of the project with the help of the project leaders and experts. 

### 2.1. CHRODIS-PLUS Implementation Strategy

**Figure 1:**
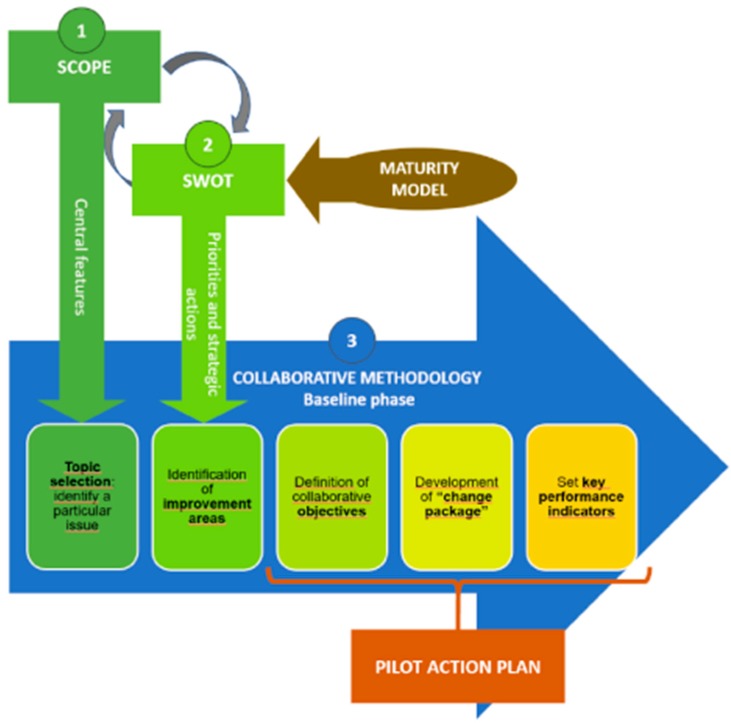
Description of the implementation phases followed by the local implementation working groups.

The implementation strategy was developed in CHRODIS-PLUS to support intervention transfer and implementation in new contexts. It was designed to be feasible enough according to the resources of the project (see Figure 1). The strategy consists of four stages that will be followed by all implementation sites [20,21] Context is defined here as the local setting, i.e., the local organization in which the implementation of a good practice takes place. The setting has its inner context with people, guidelines, decision-making structures, etc. For each case, the outer contexts also vary. It could consist of municipality, local, or national educational and/or health and/or health-promoting systems that all could affect intervention implementation. The CHRODIS-PLUS executive board, experts, and researchers are part of the outer context.

Step 1: Scope Analysis

In the scope analysis, each local implementation working group selects the specific aspects of their planned intervention. These aspects are identified according to the local context: the health situation of the local population and local needs, interests, and capabilities. A structured group discussion is used. It proceeds in five steps: (1) identify and describe the problem/challenge, (2) describe the general purpose of the intervention, (3) describe the target population, (4) analyze the intervention’s components and identify the central features that are essential to achieve the desired results, and (5) select the components from the proposed good practice that will be locally implemented. 

Step 2: SWOT Analysis

Situation analysis—“strengths, weaknesses, opportunities, threats” (SWOT)—is used to identify the respective organizations’ internal strengths and weaknesses, as well as external opportunities for, and threats to, implementing the interventions based on the selected model elements. SWOT is designed to help with both strategic planning and decision-making in relation to the planned intervention. SWOT was chosen as a tool because it is a structured, well-known, and easy-to-use method. During the SWOT analysis, the local working group in each implementing site considers the strengths, weaknesses, opportunities, and threats to the implementation of a good practice across five dimensions: (1) sustainability, (2) organization, (3) empowerment, (4) communication, and (5) monitoring and evaluation. 

A template was developed in CHRODIS-PLUS to facilitate discussion. All implementing sites prepared a document that presented the most important strengths, weaknesses, opportunities, and threats for their organization, with an overview of major issues, priorities, and strategic actions needed in relation to their planned intervention.

Step 3: Elaboration of Pilot Action Plans

The pilot action plans are developed and improved by the implementing sites during the face-to-face meetings between the members of the local implementation working groups. An action plan provides a concrete set of steps and activities that need to be conducted in order to implement their respective health promotion interventions.

An adapted version of the iterative cyclic nature of “collaborative methodology” [22] was used for developing the action plans. Following the methodology, the implementing sites addressed three main questions: (1) What are we trying to accomplish? (2) What changes can we make that will result in a successful implementation of the proposed good practice as well as improvement? (3) How will we know that a change is an improvement? These questions were used to develop a concrete action plan, which was devised in five steps: (1) identify the specific issues to work on, (2) detect improvement areas, (3) define specific objectives, (4) develop the change package, and (5) set key performance indicators. A template of an action plan was developed for the implementing sites to use. 

Step 4: Plan–Do–Study–Act (PDSA) Cycle

The plan–do–study–act (PDSA) cycle presents a pragmatic scientific method for testing changes in complex systems. The four stages mirror the scientific experimental method of formulating a hypothesis, collecting data to test this hypothesis, analyzing and interpreting the results, and making inferences to iterate the hypothesis [23,24,25]. The pragmatic principles of PDSA cycles promote the use of an iterative approach to test interventions. This enables rapid assessment and provides flexibility to adapt the intervention according to feedback to ensure fit-for-purpose solutions are developed.

The steps of the PDSA approach are as follows: (1) plan: plan the actions defined in THE PILOT ACTION PLAN TO TEST THE changes. Detail actors (who), functions and roles (what), timeframe (when) and setting (where); (2) do: test the action and, once finished, data are collected and any problem or unexpected observation is documented; (3) study: the data obtained during the testing step are analyzed. The obtained results are compared to the predictions. Learning is summarized; (4) act: based on the lessons learned changes are refined. Modifications are determined. This improved change is then re-implemented in a new PDSA cycle.

### 2.2. Protocol for Supporting the CHRODIS-PLUS Implementation Strategy

Process: We study the implementation of two good practices - Active School Flag (children in schools) and multimodal training intervention (older people) in new countries and contexts. In the first year, all partners involved reviewed and agreed on the common use of the CHRODIS-PLUS implementation framework. The good practice implementers completed a scope analysis, SWOT analysis, and an action plan. This involved recognizing the existing structures and local resources where the good practice is to be implemented. The implementers assessed and adjusted the intervention implementation to their local working culture and situation.

Roles: CHRODIS-PLUS project leaders, researchers, and good practice owners supported the new implementers, provided tools to complete implementation strategy steps, and facilitated group discussions. There was use of an external expert who commented on the pilot action plans in order to give insight and to make them more effective. Work package leaders monitored the implementation process during the implementation period. The methods used for support and monitoring were site visits and active communication through bimonthly meetings, email, and social media channels. 

Data: Documents from each implementation strategy step, notes from the meetings, site visits, recorded webinars, and other documentation from the communication were stored. This qualitative data were analyzed to gain understanding of factors associated with the implementation process.

### 2.3. The Active School Flag Implementation in Italy and Lithuania

The Active School Flag (ASF) is an Irish initiative which aims to enhance levels of physical activity for children through developing a physically active and physically educated school community. The ASF is a nationwide initiative focused on supporting a whole school approach to enhancing physical activity. The ASF mirrors other “active school” models operating throughout Europe and internationally, for example, the Centers for Disease Control and Prevention Comprehensive School Physical Activity Program in the United States (US). The target group is school-going children between the ages of five and 18 years. It is open to all primary, post-primary, special needs education schools, and youth-reach centers. Schools are recruited to the program by invitation and, once engaged with the program, they are supported on a program of action planning and self-evaluation. 

Schools are required to review their current provision across the areas of physical education (PE) and physical activity, as well as to commit to a number of improvements. The review areas include elements of planning and PE curriculum, professional development, school PE resources, activity during break times, cross-curricular and extra-curricular activity, inclusive physical activity, and active travel.

Azienda Sanitaria Locale di Collegno e Pinerolo (Italy), via the Piedmont Regional Health Promotion Documentation Center (DORS) transferred and implemented the ASF in at least two schools in the Piedmont SHE Network. This network involves 100 schools in the region and aims to promote a whole school approach to improve the health and well-being of all pupils, as well as teaching and non-teaching staff. They implemented the ASF in one school in a rural area and one in an urban area. This was based upon voluntary recruitment. Their implementation incorporates some of the Irish self-evaluation instruments in a locally specific context. The number of children involved was 330.

The Institute of Hygiene (HI, Lithuania) works with a network of Public Health Bureaus who are the main institutions promoting and initiating the implementation of public health interventions at the municipal level in Lithuania. Two of the bureaus (Klaipeda District, Klaipeda City) implemented ASF in four schools to enhance the level of physical activity of their children through the development of a physically educated school community. The number of children involved was 1468.

### 2.4. Multimodal Training Intervention Implementation in Spain, Lithuania, and Iceland

Multimodal training interventions are of special interest for older individuals, because of their high rate of disability, functional dependence, and use of healthcare resources. Multimodal training is a six-month multimodal intervention, with nutrition and health counseling on different variables, such as on functional fitness, body composition, and cardiometabolic risk factors. The participants are healthy older individuals of 71–90 years old. The intervention consists of daily endurance training (ET) and twice-a-week resistance training. This is supported by three lectures on nutrition and four on health-related topics. The ET consists of daily walking over the intervention phase. The duration of the training session increases progressively through the six-month training period.

HI (Lithuania) implemented the Multimodal Training Intervention in two municipalities (Klaipeda District, Klaipeda City) through its network of Public Health Bureaus, which are the main institutions promoting and initiating the implementation of public health interventions. They are currently implementing a cardiovascular disease prevention program in cooperation with family doctors who refer people at risk to a training program. The bureaus organized the training programs, facilitated lectures on nutrition and physical activity, and provided individual consultations to enhance lifestyle changes. The number of adults involved was 250.

El Instituto de Salud Carlos III (ISCIII, Spain), collaborating with the Aragon Institute of Research in Health Sciences, implemented the multimodal training intervention in the Aragon region. The multimodal training intervention was carried out at existing sporting facilities and community or social activity centers for over 65-year-olds in the municipality of Utebo. The target population of the intervention were residents, 65 years of age or older, with adequate conditions (not institutionalized and independent for the basic activities of daily life and of both sexes) to begin a promotion program of physical exercise. The number of adults involved was 52.

The Directorate of Health (DOHI, Iceland) implemented the multimodal training intervention in four municipalities, thereby ensuring a good mixture of villages, towns, and cities. The program was promoted in geriatric centers and local papers and it was open to everyone aged 65 and older. After each implementation phase (six months), the approach was reviewed and adapted. The training phase included daily endurance training (30 min) at least once a week with a trainer, and strength training sessions at least twice a week with a trainer. Training programs were individualized, but participants trained together as a group and received monthly lectures about nutrition, training, aging, and physiological changes. The program was implemented as an element of the Icelandic Health Promoting Community program, and the number of adults able to participate was at least 91% of the over 65 population (50,677 in 2019).

## 3. Results of the Pre-Implementation and Implementation Period: Support and Experiences

Our results on the use of the implementation framework are based on qualitative data collected during the implementation process. 

### 3.1. Preparation for Transfer and Implementation

Knowledge exchange is vital for the successful transfer of a good practice in health promotion. The sharing of experience and evidence from lessons learned enable the implementer to avoid similar missteps or to develop plans to adequately counteract them. The use of existing knowledge is used in health promotion prior to the start of the implementation because the “pre-implementation” phase involves consultation with delivery mechanisms and organizational networks [26]. For example, for a non-antagonistic area of health promotion such as physical activity, there are frequently existing structures and experiences related to the delivery of similar programs. This means that existing staff and organizational networks are in place to facilitate the program implementation within a foundation that will support the new program [12]. In this case, we found that brief but substantive information is required by the new implementers who can comprehend the new program fitting in within existing programs (see Section 3.3 on embedding within existing programs). In contrast, in cases in which elements of health promotion are less well established, more extensive pre-implementation consultation is required. 

In CHRODIS-PLUS, we conclude that knowledge exchange was key to all implementers. We organized knowledge exchange in the form of site visits. This is where the implementers of the good practice visit the good practice in situ. This enables good practice implementers to become more familiar with the implementing site, and they are, thus, better able to provide advice that suit the local needs. These site visits were identified as crucial for the implementing of the good practice (see Table 1). 

The multimodal training intervention implementation relies on specific technical capacities of the implementers. This involves testing strength, blood, and other corporeal capacities. This is an example of the need of more extensive pre-implementation knowledge exchange, achieved through site visits and a return visit from the good practice owner to the field site where implementation is being conducted (we return to this in the discussion). Site visits were organized between multimodal good practice owners from Iceland and implementers in Lithuania and Spain.

The Icelandic partners (Janus Health Promotion) had the opportunity in both of these countries to take a look at the training facilities and help out with fitness level measurement-days. They also gave lectures for the participants (in Lithuania) and had a meeting with decision makers in Klaipeda District Municipality and decision-makers in Utebo-Zaragoza in Spain. During and after the visit, the Icelandic partners developed a set of recommendations for the implementers in Lithuania and Spain for further improving the physical training of this good practice project for those aged over 65.

Site visits between the Irish good practice owners of ASF and the implementors in Italy were conducted in June 2018 in Ireland and in October 2019 in Italy. Based on the experience from the owners of the good practice and implementers, these site visits were key moments to enter the process, to share experiences, and to receive suggestions and directions from donors. They recognized that there is a need for one site visit to implementers at the early stage of the process for the understanding of the specific features and characteristics of the context and one at the middle/end of the process to redirect activities and introduce any changes/additions to the implemented activities. A missed opportunity involved the unforeseen moments of connection, comparison, and reflection between different implementers sites (Italy and Lithuania). For example, specific meetings with implementers and donors would have been enriching for everyone.

### 3.2. Engaging Those Who Deliver Health Promotion Programs 

Communication was found to be a central element of the successful transfer and implementation of health improving programs [27]. In our work, in CHRODIS-PLUS, we made active communication a corner stone of how the transfer of programs happens. 

Webinars and meetings with topics like the different implementation strategy methods were organized during the implementation phase for the implementers of the good practices in different countries. The implementers and the good practice owners held bimonthly meetings during the implementation period. Meetings were held with videoconferencing systems; thus, they were easy to attend in different countries. A template for reporting the progress was developed. It was filled in by the good practice implementors regularly. Not every group was able to hold meetings as often as first planned (i.e., every other month), but there were regular meetings, with at least three or four each year per implementing site. There were some challenges in finding a suitable video-conferencing system for everyone and also finding time when working in different time-zones. In the future, when designing implementation projects, one should take into account working connections and agree beforehand on which video-conferencing system is used and also plan a budget for it. In the template, is the following short questions were asked:

- What were you doing during this month?
- What was recorded (quantitatively or qualitatively)?
- What were the successes?
- Were there any barriers?
- Was any support needed?
- What are you planning to do next month?
- What was the assessment during the current period (quantitatively or qualitatively)?
- What were the perceived barriers?
- How certain are you that you will achieve what you are planning to?

The good practice owners also kept contact with the implementors through emails. For example, multimodal training good practice implementors also had a closed Facebook group for sharing. In the Facebook group, it was very easy to share photos of the local implementation groups and messages of the challenges and successes. The use of new channels of communication eases the burden on language skills and also helps to spread success stories that might not be reported in any other way. 

### 3.3. Embedding a Program within Structures and Resources

An aspect of the implementation strategy that must not be overlooked is the recognition of a problem. In the implementation strategy, the use of scoping analyses enables implementing organizations to assess their current activities, workload, and resources. It also involves the identification of potential facilitators and barriers. As highlighted in the literature on implementation science, these are key elements in the successful transfer of a new program [4]. They are indeed vital to ensure that the local context is suitable and able to support any new intervention. However, human resources are also vital for any intervention. Again, this can often be overlooked when thinking about what to implement, how to implement, and how to report on any implementation [28]. Not only do human resources need to be accurately measured and considered, but, without the buy-in of key people, any implementation will struggle. 

In the implementation projects that we conducted in CHRODIS-PLUS, the identification and support of influential decision-makers was important. We found, through the implementation framework that includes assessing barriers, facilitators, and threats, that active support by senior figures is necessary but must extend deeper than written policies. This is because the organization and delivery of health promotion programs can be experienced as an additional responsibility for those involved in the implementation. Those at the delivery end are unlikely to want extra burden if it is perceived as risky for their professional life, personal well-being, or work–life balance. The pathway of program introduction and delivery needs to be both paved (practical assistance—specific training, resources, and co-ordination with other aspects of their work) and sheltered (from local or national outside parties who disagree with a program’s focus or approach) [26].

### 3.4. Affinity of the Program with Current Practice and Interests

Local context is key, and this was emphasized in the literature [10,11]. This worked two ways in our implementations of the Active School Flag in Italy and Lithuania. Firstly, embedding the good practice within an environment where a health-promoting school concept was already in place provided fertile ground in terms of network and support. The motivation of those delivering programs to engage in training that addressed knowledge or skill deficits was more likely if they had experience with such a program. In this case, schoolteachers and support staff were more likely to engage when they could see the likely personal, social, and developmental gains from participating. The use of a good practice to expand (or “mesh with”) a current program aids transfer and implementation [26,29]. 

Secondly, the addition of a new factor to a good practice feeds back into the buy-in of senior decision-makers and local concerns [30]. For example, the use of a healthy school program with the addition of a dental care element both expands the implemented program and develops a locally specific hook that encourages participation of the program and uptake. In some cases, even where there appears to be a lack of concordance between a program and some activities, this can act as a stimulus for change and mutual accommodation. However, this will require early recognition and careful pre-implementation assessment, as set out in the implementation framework using SCOPE and SWOT analysis, which we introduced in the methods section.

## 4. Discussion 

The findings from our implementation projects suggest that the transfer of a good practice is not straightforward. We established that attributes of the good practice, preparation for transfer, and program affinity play an important role in the process. The decision to implement an existing good practice is an active and dynamic process. 

Our study supports the literature that argues that the effectiveness of implementations is critically influenced by their given context [10,13]. In order to mitigate factors associated with context (e.g., social, cultural, political, and institutional setting), a clear framework that enables implementers to assess their local health system/setting context was important for the implanting sites in this study [11,12]. The implementation framework we have described involved SCOPE and SWOT analysis to enable a comparison of information to identify differences and similarities across sites. The collaborative methodology encourages groups to come together to share their knowledge and ideas on their chosen area for improvement [22].

We identified essential replicable mechanisms that impact the successful transfer and implementation of a health promotion program. An element that was important in our implementing sites was the need for an amount of conformity between current activities and the proposed health promotion program [10,14,26]. A key replicable mechanism is to foster active communication between good practice owner and implementing site. In our study, this was achieved by site visits and structure bi-monthly video calls. In addition, local hooks that acted as a connection between new programs and existing programs helped to ease transfer and encourage participation by key decision-makers [29].

We found that it was essential to have participation by key decision-makers throughout the process of preparing for transfer and implementation [30]. However, while it is important that implementation is a process that needs key decision-maker involvement, it is essential that the process is overseen by administrators of change coupled with a clear implementation framework [31]. Administrators of change are people who can bridge the divide between outcomes and different stakeholder groups [32]. During the transfer and implementation process, it is essential to identify administrators of change. These are people who offer support to initiatives through their vision of the wider picture. Administrators of change can guarantee that a program or practice has probity and constancy for the context within which it is implemented. Administrators of change can be conducive in ensuring that colleagues and those delivering the implementation will persist even when confronted with problems during the process.

Environmental and individual barriers that prevent the successful transfer of good practices include a lack of peer support, competition with other programs (saturation at schools for the Active School Flag is a particular concern), and staff turnover (see Table 1). The barriers were experienced by the programs that were implemented within CHRODIS-PLUS. An interesting finding of the implementation so far is that engaging those who deliver health promotion programs through social media is a new technique that helps to support people implementing programs and it is a new area that requires further study for implementation science (this was the case for the implementation of the multimodal training program in Spain and Lithuania). This is because contextual factors and challenges can be assessed and mitigated with the aid of the good practice owner and other implementers experiencing similar problems. 

There are several strengths of the implementation strategy [21]. We applied a framework that followed a standardized procedure that each local site followed and used to transfer according to their attributes, capacity, and requirements. The framework was organized and refined in order for each site to follow the same methodology to create a standardized implementation package that could be practically applied in different health promotion settings. Each site participated in regular meetings with the project coordinator to compare strategies and identify and mitigate any potential deviations in the methodology. In addition, we used tools such as SWOT and SCOPE analysis to provide a comparison of information to identify differences and similarities across sites [20].

A limitation of the study we presented in this paper is that we focused on implementations that promote physical activity, and there could be different challenges facing different types of programs when they are implemented. Moreover, the implementation process was limited to 12 months so as to align with the timeframe of the three-year CHRODIS-PLUS joint action. Therefore, various aspects might reflect this relatively short intervention period [21]. However, our findings are from projects that were implemented in significantly different contexts (e.g., an Irish program in Lithuania and Spain) and, therefore, represents a worthwhile addition to the literature on health promotion implementation.

## 5. Conclusions

In this paper, we ascertained some key features of successful transfer of good practices. Firstly, it is essential to develop an action plan of key activities and to plan carefully how the support of the implementation is organized during the implementation period. Secondly, embedding the good practice within existing structures and resources is crucial, as is recognizing the specific characteristics of each local context where the good practice is to be implemented. Thirdly, site visits during the implementation period were found to be extremely useful both to the implementers of the good practice and the good practice owners. After a site visit, it is easier to guide the implementation since the knowledge of the specific issues in the local context is better understood and key relationships are established. However, in future implementation research, a clear budget for translation and travel is required. This is of particular importance when implementing programs in a country with a different language and regulatory environment. 

Our research into the transfer of good practices helps to develop the knowledge base for health promotion and disease prevention activities. In doing so, it develops our understanding of how health promotion works in different contexts and the health promotion landscape within Europe [33]. However, it is clear that more research is required to fully comprehend the difficulties in the transfer of good practices from one context to another. Our work with colleagues from across the Joint Action CHRODIS-PLUS will go some way to answering these difficult questions, and it will help to improve health and well-being for all.

## Figures and Tables

**Table 1:** Table of barriers and facilitators for transfer and implementation.

Implementing Site	Good Practice	Barrier	Facilitator	Additional Factors
Piedmont Regional Health Promotion Documentation Centre, Italy	Active School Flag	1. Lack of human resources/staff turnoer 2. No local hook for programme to connect 3. Lack of peer support 4. Competition with other programmes	1. Site Visit 2. Existing knowledge 3. Bi-monthly meeting and reporting template 4. Senior decision makers buy-in 5. Embedding the good practice within an environment where a similar programme is already in place 6. Locally specific hook that encourages participation of the programme and uptake	1. Active support by senior figures must extend deeper than written policies
Klaipeda District Public Health Bureau, Lithuania	Active School Flag	1. Lack of human resources/staff turnover 2. No local hook for programme to connect 3. Lack of peer support 4. Competition with other programmes	1. Site Visit 2. Existing knowledge 3. Bi-monthly meeting and reporting template 4. Senior decision makers buy-in 5. Embedding the good practice within an environment where a similar programme is already in place 6. Locally specific hook that encourages participation of the programme and uptake	1. Active support by senior figures must extend deeper than written policies
Klaipeda City Public Health Bureau, Lithuania	Active School Flag	1. Lack of human resources/staff turnover 2. No local hook for programme to connect 3. Lack of peer support 4. Competition with other programmes	1. Site Visit 2. Existing knowledge 3. Bi-monthly meeting and reporting template 4. Senior decision makers buy-in 5. Embedding the good practice within an environment where a similar programme is already in place 6. Locally specific hook that encourages participation of the programme and uptake	1. Active support by senior figures must extend deeper than written policies
Aragon Institute of Research in Health Sciences, Spain	Multimodal training intervention	1. Lack of human resources 2. No local hook for programme to connect 3. Lack of peer support	1. Site Visit 2. Return visit from the good practice owner 3. Bi-monthly meeting and reporting template 4. Senior decision makers buy-in	1. Closed Facebook group for sharing photos of the local implementation groups and messages of the challenges and successes
Klaipeda District Public Health Bureau, Lithuania	Multimodal training intervention	1. Lack of human resources 2. No local hook for programme to connect 3. Lack of peer support	1. Site Visit 2. Return visit from the good practice owner 3. Bi-monthly meeting and reporting template 4. Senior decision makers buy-in	1. Closed Facebook group for sharing photos of the local implementation groups and messages of the challenges and successes
Klaipeda City Public Health Bureau, Lithuania	Multimodal training intervention	1. Lack of human resources 2. No local hook for programme to connect 3. Lack of peer support	1. Site Visit 2. Return visit from the good practice owner 3. Bi-monthly meeting and reporting template 4. Senior decision makers buy-in	1. Closed Facebook group for sharing photos of the local implementation groups and messages of the challenges and successes
Directorate of Health, Iceland	Multimodal training intervention	1. Lack of human resources 2. No local hook for programme to connect 3. Lack of peer support	1. Site Visit 2. Bi-monthly meeting and reporting template 3. Senior decision makers buy-in 4. Inter-sectoral collaboration and consultation at the national level being strengthened with consultation from implementing sites	1. Closed Facebook group for sharing photos of the local implementation groups and messages of the challenges and successes 2. Good practice owner is based in and the good practice was developed in Iceland (re: point 4—facilitators).
